# New and Recovered Temporary Anchorage Devices, In Vitro Assessment of Structural and Surface Properties

**DOI:** 10.3390/ma14216271

**Published:** 2021-10-21

**Authors:** Paula Argentina Jiman, Doina Prodan, Marioara Moldovan, Alexandrina Muntean, Codruta Sarosi, Viorica Tarmure, Grigore Baciut, Catalin Popa, Andreea Simona Pop

**Affiliations:** 1 Faculty of Dentistry, Iuliu Hatieganu University of Medicine and Pharmacy, 31 A. Iancu Street, RO-400083 Cluj-Napoca, Romania; paula.jiman@umfcluj.ro (P.A.J.); viorica.tarmure@umfcluj.ro (V.T.); m.baciut@umfcluj.ro (G.B.); andreea.pop@umfcluj.ro (A.S.P.); 2 Faculty of Materials and Environmental Engineering, Technical University of Cluj-Napoca, 103-105 Bd. Muncii, RO-400641 Cluj-Napoca, Romania; catalin.popa@stm.utcluj.ro; 3 Institute of Chemistry Raluca Ripan, Babeş-Bolyai University, 30 Fantanele Street, RO-400294 Cluj-Napoca, Romania; doina_prodan@yahoo.com (D.P.); mmarioara2004@yahoo.com (M.M.); codruta.sarosi@gmail.com (C.S.)

**Keywords:** orthodontic miniscrew, surface morphology, surface treatment, SEM, titanium alloy

## Abstract

The orthodontic miniscrew (TADs) is a device that is fixed into bone in the short term for the purpose of enhancing orthodontic anchorage. The aim of our study was to investigate the structural and surface properties of recovered TADs after orthodontic treatment, and compare them to new TADs. TADs (n = 15) from the same manufacturer (Absoanchor; Dentos, Daegu, Korea) were assessed; n = 10 were recovered from patients after orthodontic treatment and n = 5 were new. We performed electrochemical investigations, scanning electron microscopy (SEM) and microbiological analysis. Qualitative analysis on general electrochemical polarization revealed that the TADs retrieved from the patients provided much lower current densities in the passivity zone, and the oxidative processes taking place on their surface were of lower intensity. The surface morphologies of the tips of the retrieved mini-implants showed less sharp tips and smooth surfaces. Defects in the form of pores or cracks could be identified in both evaluated TAD groups. All retrieved TADs showed signs of biological materials (SEM analysis) and contamination on their surfaces. In conclusion, these results can assist orthodontists in comprehending the complexities of TAD behavior with respect to their design and structure.

## 1. Introduction

Orthodontic miniscrews were first made known by Gainsforth and Higley in an animal study. A Vitallium screw was placed in the anterior edge of the mandible branch of five dogs, to apply traction using orthodontic elastic connected to a maxillary device for skeletal anchorage [1]. In 1997, Kanomi first described the “mini-implant”, designed specifically for orthodontic applications [2]. A temporary anchorage device (TADs) is a device that is, in the short term, fixed to the bone orthodontic anchorage supporting the teeth of the reactive unit, or avoiding the need for the reactive unit, and which is removed after use [3,4]. An implant is defined as an alloplastic device that is surgically inserted into or onto the jaw bone. Commonly used TADs consist of three parts, as follows:

- Implant head: serves as an element for the positioning of orthodontic accessories;
- Transmucosal neck;
- Implant body: the part embedded inside the bone.

In the last two decades, titanium mini-screws have gained enormous popularity in orthodontics and are often considered as a source of absolute intraoral anchorage, extending the possible aims and limits of orthodontic treatment. The possibility of applying immediate loading represents another advantage, reducing the total duration of orthodontic treatment [5,6].

Compared to other anchoring forms, TADs offer more predictable results. TADs are made of an alloy of pure commercial titanium and grade V titanium, which is favored due to its higher strength compared to pure commercial titanium. Contemporary TADs are designed to be easy to insert and are generally safe to use. However, miniscrews have been reported to occasionally cause gingival injury and fractures, due to mechanical breakdown in the oral environment. Failed TADs require removal and/or replacement and influence the success of orthodontic treatment; careful patient selection, implant site characteristics, macrostructure, microstructure and mechanical properties are key elements in achieving adequate results [7,8].

When an implant is placed in the bone, mechanical stress, corrosion or cracking of the alloy can occur. Although titanium alloys are known to be exceptionally corrosion resistant due to the stability of the passive layer of titanium oxide on the surface, TADs have been reported to suffer from corrosion after clinical application [9,10]. Cracking arises between two nearby surfaces or in confined spaces, where oxygen exchange is not available and can spread in physiological or corrosive environments [10].

Once the necessary orthodontic goals have been achieved, the TADs are removed from the patient’s bone, and are usually discarded. Economic or environmental factors could influence clinicians in considering reusing TADs, especially in patients who have lost their miniscrews or where the mechanical requirements of orthodontic treatment impel different positioning of the anchorage device. Not all orthodontic miniscrews can be used again, but those made of titanium alloy are easier to reuse because they can be mechanically and chemically cleaned and resterilized with little or no loss of shape or function [11].

With the aim of obtaining a better understanding of clinical behaviors, several studies have described the surface characteristics and mechanical properties of the recovered orthodontic TADs [12]. Eliades et al. [10] identified morphological and structural changes when assessing TAD hardness following recovery after orthodontic treatment; they reported that TADs made of titanium alloy exhibited morphological and structural surface changes. Mattos et al. [13] compared received, sterilized and recovered mini-implants, assessing the fracture torque risks of reusing these devices after sterilization. According to these authors, no defects or corrosion could be identified after sterilization of the recovered mini-implants in an autoclave, but worn surfaces and scratches were observed.

Sebbar et al. evaluated the surface changes of TADs recovered after use in comparison with new TADs under an optical microscope [14]. Recovered TADs exhibited signs of corrosion, predominantly at the site of manufacturing defects [14].

TADs offer several advantages, including interdental placement, simplified and less traumatic surgery, faster healing, and immediate orthodontic load. Specific TAD parts come into contact with various elements of the oral cavity. The head and neck regions come into contact with the oral mucosa, along with saliva and food debris, thus encountering mechanical and chemical stress. TAD body parts come into contact with the alveolar bone and ensure the stability of the mini-screw. It is essential that the structural and mechanical properties are maintained until the TADs are removed [15,16].

The aim of our in vitro study was to investigate the structural and surface properties of recovered TADs after orthodontic treatment, in comparison with new TADs treated in different storage media. The null hypothesis was that exposure time, type of alloy, and type of storage medium would influece the corrosion performance of orthodontic miniscrews.

## 2. Materials and Methods

This study was conceived under a protocol approved by the University of Medicine and Pharmacy “Iuliu Hatieganu” (Cluj-Napoca, Romania) Ethics Committee (decision nr. 221/17 May 2017).

For this in-vitro study, 15 TADs, 8 mm long, 1.5 mm in diameter, console head type, self-drilling, made of grade V titanium alloy were used. All of the investigated TADs were from the same manufacturer (Absoanchor; Dentos, Daegu, Korea) in order to prevent any bias related to device design and material properties.

Patients selected for TAD placement presented Angle Class 1 bimaxillary protrusion, diagnosed in accordance with the following criteria: facial convexity, lip incompetence, dentoalveolar protrusion, molar class I occlusion, 4 mm overjet, overbite and more than 5 mm crowding in upper and lower arch. For all patients, treatment plan consisted of the extraction of four first-premolars and then the alignment and retraction of canines and incisors.

TADs were placed for anchorage support in the buccal area during the alveolar process, on the attached gingiva (insertion height at 7 mm away from the interdental papilla) and perpendicular to the buccal cortical bone, between the 1st molar and 2nd premolar in the upper and in the lower arch. The miniscrews were placed at 45° angles relative to a line perpendicular to the occlusal plane.

Titanium miniscrews (Absoanchor, Dentos, Daegu, Korea), 1.3 mm in diameter and 8 mm in length, were inserted by a single operator using the following sequence:

Preliminary panoramic and periapical radiograph to estimate the angulations of the roots of the adjacent teeth.

Anesthetize the area with local anesthesia (Opahl-benzocaine 20%, Xylitol si Vitamine E, Iolite, Miami, FL, USA).

The insertion area was washed with Curasept ADS^®^ 220 with 0.20% chlorhexidine (Curaden, Kriens, Switzerland).

The screw hole was made with a 1.0 mm round bur and a drill (diameter 1.1 × 5.0 mm) at 500 rpm.

The miniscrews were inserted manually, using the Long Hand Driver (LHD-B) and Driver Tip (DT-S) provided by the manufacturer. They were inserted in the attached gingivae with the insertion angle directed apically (about 45°). The angle of insertion was assessed visually with the intent of standardizing it for all patients [17].

First, premolars postextractional spaces were closed using sliding mechanics on rectangular 0.019″/0.025″ stainless steel wires. The en masse retraction was accomplished using 9 mm NiTi coil spring attached anteriorly on hooks welded to the mesial of the canines and posteriorly to the TADs. The NiTi coil spring delivered 200 g of continuous force without any permanent deformation. Retraction was completed in 10 months.

At the end of the orthodontic treatment, in order to ensure stability of results, fixed retention with multistranded stainless steel wire was used for the mandibular arch, and a thermoformed retainer was used for the maxillary arch [18].

Ten TADs were recovered from patients after orthodontic treatment objectives were achieved and 5 TADs were new. All recuperated TADs were placed for anchorage support in the buccal area of interradicular bone between the second premolar and first permanent molar. These recovered TADs were used in 8 patients (3 male, 5 females; mean age 16 years) Recovered TADs (n = 10) were treated in different manners: sterilized (n = 5, using Vacuklav 23+, Melag, Germany) and non-sterilized (n = 5).

- Electrochemical Investigations

Electrochemical investigations were performed in order to determine the influence of the electrochemical and thermochemical surface treatment on the anticorrosive performances of the anodic oxidation layers on recovered and new TADs. The analysis was qualitative and comparative, with the anodic layer, not thermochemically processed, being used as the reference. The rationale of this analysis was to assess the influence of thermochemical processing on the electrochemical performance of the anodic layers, in order to improve their performances.

The experimental technique used was cyclic voltammetry. We worked with a potentiostat PS4-MLW (IPS Elektroniklabor GmbH & Co. KG, Münster, Germany), connected to a PC through a PROSYS interface. The following sampling and storage parameters were set for the laboratory experiments: sampling time 1000 × 100 s, 0.1 mA current scale head. The surface investigated on the samples consisted of the entirety of the TADs. The electrolyte used was Ringer’s solution, with a pH of 2.5 and a 1:1 citric acid: phosphoric acid ratio. The 25 °C electrolyte temperature was selected for technical reasons, as it was the laboratory temperature. The potential range investigated was 1500–2000 mV/s.

The polarization speed in this case was 100 mV/s, which is at the lower limit of the working speeds in cyclic voltammetry. A total of 10 and 20 complete cycles were performed for all samples, and the results were recorded, evaluated and represented in distinct figures.

- Scanning Electron Microscopy Investigation (SEM)

Before analysis using a scanning electron microscope, the specimens from the retrieved group were submitted to a cleaning cycle of 30 min in a Codyson CD-3800A ultrasonic cleaner (Codyson Electrical Co. Ltd., Shenzhen, China) and rinsed. Cleaning solution was prepared using 1 L distilled water and 5 mL Endozyme (Ruhof Co., Long Island, NY, USA). Then, each miniscrew was packed separately, sterilized at 135 °C for 10 min and dried for 55 min. Digital images were acquired by SEM before and after in vitro TAD processing. Evaluation by scanning electron microscopy (SEM) was performed by a single examiner using INSPECT S (FEI Company, Hillsboro, OR, USA) at the Institute of Chemistry “Raluca Ripan”, Cluj-Napoca, Romania.

- Microbiological Analysis

Microbiological analysis was performed using: *Streptococcus mutans* ATCC 25175 and *Porphyromonas gingivalis* ATCC 33277 from the collection of the Laboratory of Microbiology, Faculty of Biology and Geology, UBB, Cluj-Napoca, Romania. The culture media used were nutrient agar and TSB (trypticase soy broth) (Atlas, 2010). Sterile petri dishes, 1.5 mL sterile Eppendorf tubes, sterile micropipettes and tips, sterile tweezers, a Consort pH meter, a BIO-M SCS 1–4 vertical air laminar hood (Cruma – Diantech Solutions S.L., Barcelona, Spain), an electric Raypa autoclave (Raypa, Barcelona, Spain), and a Salvis IC400 (SalvisLab Industrie-Ost, Rotkreuz, Switzerland) incucentric incubator were used.

Microbiological method. Each bacterial strain was grown for 24 h on nutrient agar medium. A colony was then isolated from each strain and inoculated on liquid TSB culture medium to obtain the bacterial suspension. In the Eppendorf tubes inoculated with the test strains, TADs were transferred and then incubated at 24 h at 37 °C. Subsequently, TAD surfaces were analyzed using SEM at different magnifications in order to detect the formation of bacterial bio film [12,19].

## 3. Results

### 3.1. Electrochemical Evaluation

**Figure 1:**
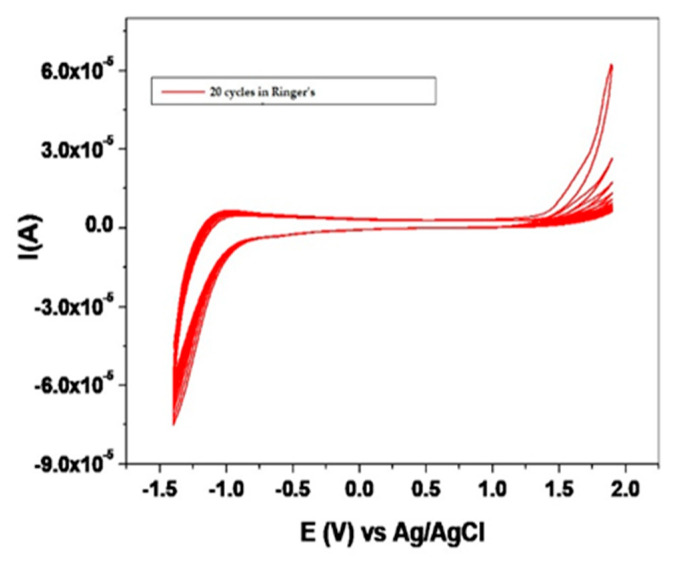
Voltammograms of 20 cycles in Ringer’s solution, temperature 25 °C, with a speed of 100 mV/s, for retrieved (patient 1) and new mini-implants.

**Figure 2:**
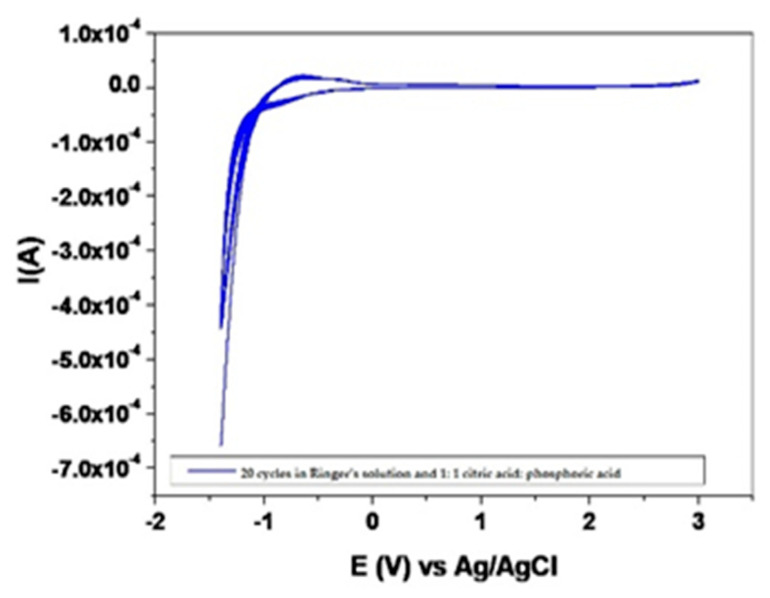
Voltammograms of 20 cycles in 1:1 citric acid: phosphoric acid solution, temperature 25 °C, with a speed of 100 mV/s, for retrieved (patient 1) and new mini-implants.

The analysis performed in this study was a qualitative analysis of the general electrochemical polarization of the samples, over four cycles. All experimental samples showed a spontaneous passivation in the electrolyte used as a result of irreversible anodic reactions and a stable state of passivity. Figure 1 and Figure 2 show the voltammograms for the retrieved and new TADs with 20 cycles in Ringer’s solution and 1:1 citric acid: phosphoric acid solution, at 25 °C temperature, with a speed of 100 mV/s. The state of passivity is accentuated during anodic polarization; the polarization cycles located below the cycle 1 path, present a successively lower hysteresis. Figure 1 presents the polarization cycles for the recovered and sterilized TADs (patient 1) and the reference sample.

Figure 2 presents the polarization cycles for the recovered and sterilized TADs (patient 1) and the reference sample.

### 3.2. Scanning Electron Microscopy (SEM) Investigation

**Figure 3:**
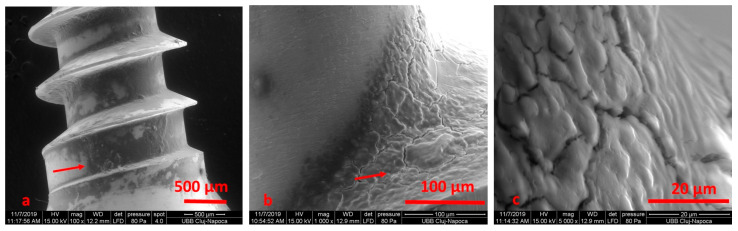
SEM micrographs of the surface screw, removed after 10 months, at ×100 (**a**), ×1000 (**b**) and ×5000 (**c**) magnification.

SEM analysis was performing on the retrieved TADs, either subjected or not to electrochemical assessment. We noticed an area of osseointegration and a regular shape for the TADs (Figure 3).

**Figure 4:**
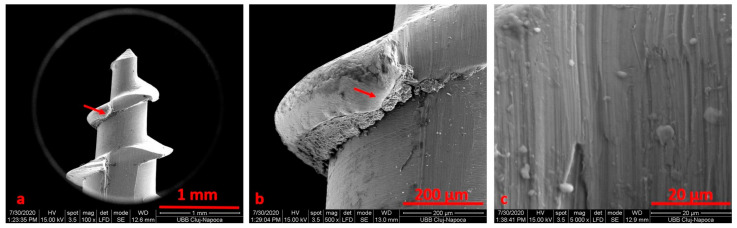
SEM image for the TADs (patient 1) before electrochemical treatment using Ringer’s Solution at ×100 (**a**), ×500 (**b**), ×5000 (**c**) magnification.

**Figure 5:**
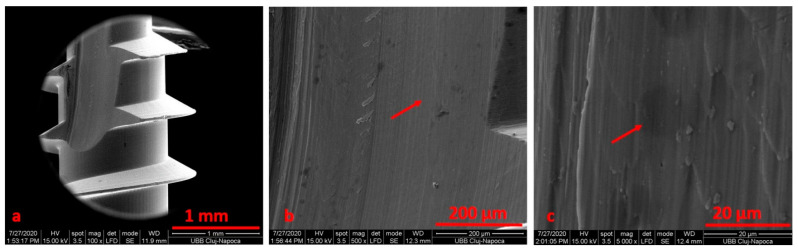
SEM image for the TADs (patient 1) after electrochemical treatment using Ringer’s Solution at ×100 (**a**), ×500 (**b**), ×5000 (**c**) magnification.

**Figure 6:**
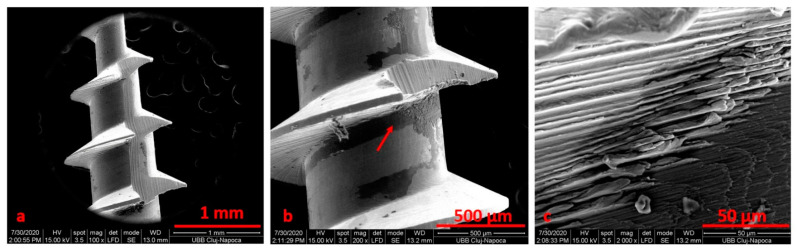
SEM image for the TADs (patient 2) before electrochemical treatment using 1:1 citric acid: phosphoric acid solution at ×100 (**a**), ×200 (**b**), ×2000 (**c**) magnification.

**Figure 7:**
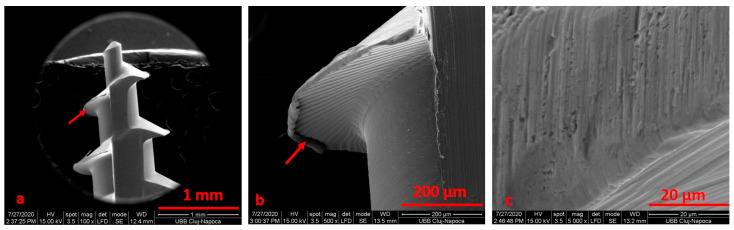
SEM image for the TADs (patient 2) after electrochemical treatment using 1:1 citric acid: phosphoric acid solution at ×100 (**a**), ×500 (**b**), ×5000 (**c**) magnification.

After electrochemical treatment, the surface appeared smooth, but we noticed pits and cracks. The retrieved mini-implant scratch marks suggest that insertion and removal may have resulted in wearing. After cleaning, we noticed an oxidation area (black) on the surface (Figure 4, Figure 5, Figure 6 and Figure 7).

**Figure 8:**
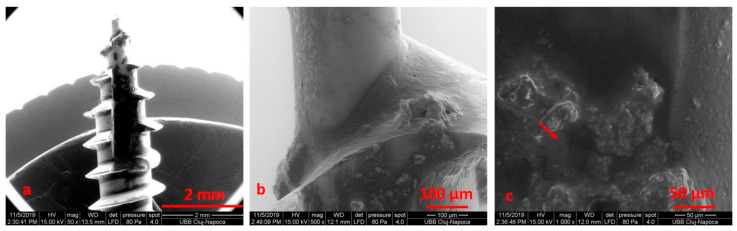
SEM images of non-sterilized mini-implants, recovered from patients, at ×50 (**a**), ×500 (**b**), ×1000 (**c**) magnification.

**Figure 9:**
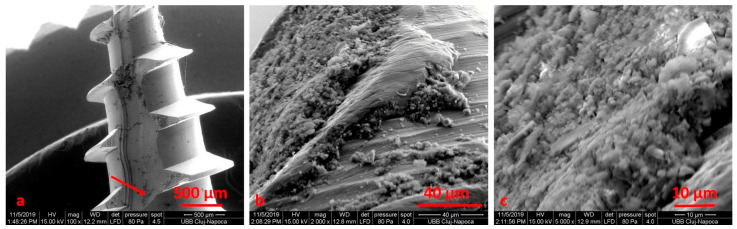
SEM images of sterilized mini-implants, recovered from patients, at ×100 (**a**), ×2000 (**b**), ×5000 (**c**) magnification (cracks).

We noticed that the tip of the retrieved mini-implant showed a smooth surface and a less sharp end (Figure 7a,b). Defects in the form of pores (Figure 8c) or cracks could be identified in both the sterilized and non-sterilized TAD group (Figure 8 and Figure 9).

The used miniscrew exhibited indentation, corrosion attacks and cracks over the entire surface. No defects in the form of pores or cracks and no images suggestive of corrosion could be identified in the autoclaved as compared to the as-received mini-implants.

### 3.3. Microbiological Analysis

**Figure 10:**
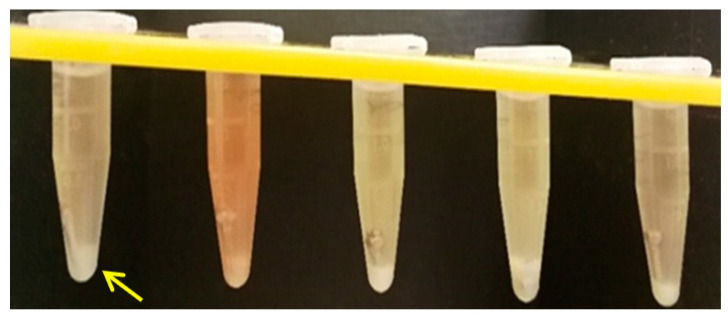
Bacterial proliferation—*Porphyromonas gingivalis* (PG) culture.

**Figure 11:**
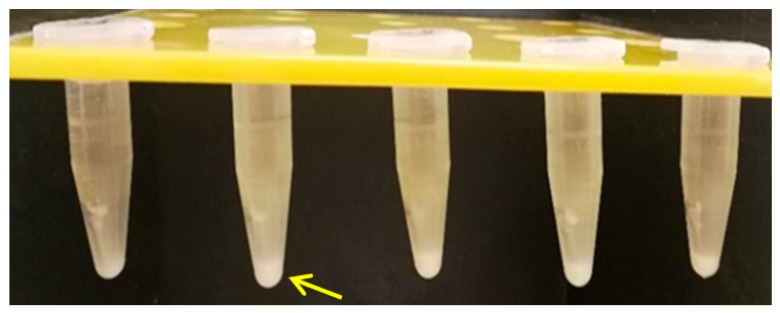
Bacterial proliferation—*Streptococcus mutants* (SM) culture.

The retrieved TADs showed the precipitation of an amorphous layer, more consistent for PG (Figure 10) when compared to SM (Figure 11).

**Figure 12:**
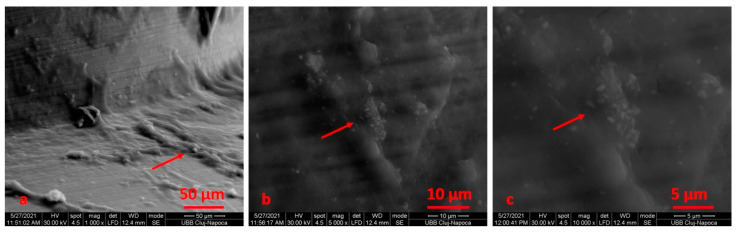
SEM images with mini-implants and PG at ×1000 (**a**), ×5000 (**b**), ×10,000 (**c**) magnification. Extensive bacterial colonization (**a**–**c**) on the mini-implant bodies.

**Figure 13:**
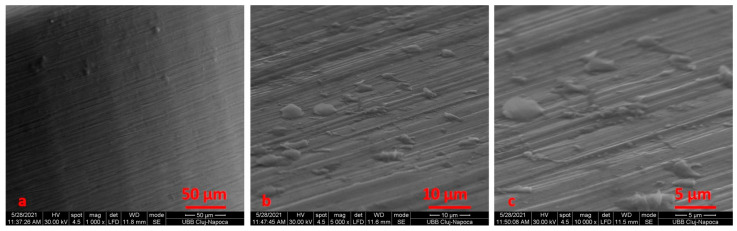
SEM images with mini-implants and SM at ×1000 (**a**), ×5000 (**b**), ×10,000 (**c**) magnification. Extensive bacterial colonization (**a**–**c**) on the mini-implant bodies.

**Figure 14:**
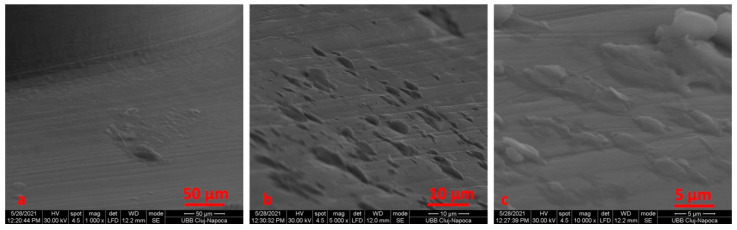
SEM images with mini-implants and SM at ×1000 (**a**), ×5000 (**b**), ×10,000 (**c**) magnification. Bacterial colonization on the mini-implant bodies (**a**–**c**).

**Figure 15:**
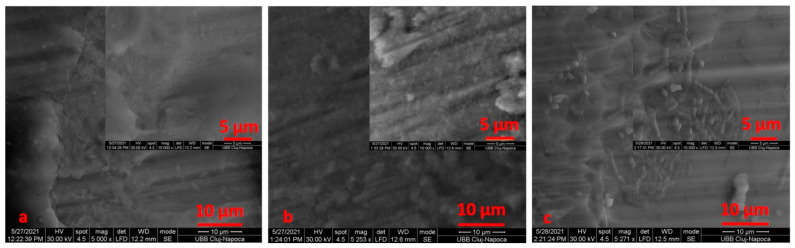
SEM images that highlight the formation of bacterial colonies on the screw surface: (**a**) new and untreated; (**b**) after electrochemical treatment using Ringer’s Solution; (**c**) after electrochemical treatment using 1:1 citric acid: phosphoric acid solution.

All retrieved TADs showed signs of biological contamination on their surfaces, and the SEM analysis indicated the presence of biological materials on the miniscrew surfaces. Extensive bacterial colonization on the mini-implant bodies was noticed (Figure 12, Figure 13 and Figure 14). Figure 15 reveals specific details of the biofilm: Figure 15a,c highlight bacteria in rod form; in Figure 15b, bacteria can be seen in the form of shells, grouped as clusters.

## 4. Discussion

The surface characteristics of the TADs influence the interface with osseous tissue and microbiological behavior.

The biocompatibility of TADs is a determining factor, as they are inserted directly into periodontal tissues and alveolar bones. Adverse reactions (inflammation, necrosis of the oral, gingival, or alveolar mucosa [19,20]) are strongly correlated with the stability of the alloys used to produce TADs, as metal ions released can interact with the complex environment of the oral cavity [20].

### 4.1. Electrochemical Evaluation

TADs retrieved from the patients show improved behavior compared to the reference alloy; they provide much lower current densities in the passivity zone, and the oxidative processes that take place on their surface are of lower intensity, due to the presence and characteristics of the oxide barrier. The described order of the samples persists at the superior extremity of the investigated potential domain, except for an inversion that appears between the samples tested in the two solutions. The electricity density in this area is related to the oxygen overvoltage discharge, a secondary aspect in our analysis. Double anodizing processing has beneficial effects on the alloy, increased passivity, and probably better continuity of the dielectric or semiconductor oxide layer, thus preventing the development on the surface of the samples of intense oxidative processes. This oxide film is a strong and stable layer, and prevents the diffusion of oxygen from the environment, thus ensuring corrosion resistance [21,22].

Corrosion is defined as the process of interaction between a solid material and its surrounding environment, resulting in the loss of the structural integrity, alteration in structural features, loss of substance and degradation of the material into its constituent atoms, due to the occurrence of chemical reactions [17,23]. In the oral cavity, corrosion is induced by metal ions released during orthodontic treatment by specific elements of fixed appliances [17,24].

The titanium dioxide layer of TADs can be disturbed by chemical and mechanical attacks [25,26]. Saliva is an electrolytic solution and contains acids produced by bacteria and fungi and can result in the corrosion of mini-screws. TiO_2_ is chemically stable and inert and is ideal for insertion into bone [13]. TiO_2_ has photo-catalytic properties (in its anatase form) that prevent the colonization of pathogenic microorganisms [27]. This TiO_2_ layer, also called the passivation layer, exists in all TADs tested in this experiment. By changing the anodizing voltage, the color and thickness of the anodizing layer also change. As the anodizing voltage is increased, the thickness of the anodizing layer increases. The layer of titanium dioxide on the surface of the TADs is linked both directly and indirectly to the release of toxic metal ions [13,26].

During orthodontic treatment, TADs will interface with hard and soft tissues, as well as a variety of solutions (saliva, blood, interstitial fluid, beverages, dental pastes, rinses, and gels). These changes occur because the concentration of chloride ions in the interstitial fluid, as well as the salivary assortment of amino acids and proteins, produces a corrosive environment for metallic materials. The dietary intake of a particular element may vary as a result of individual consumption, as well as geographical location, but studies have shown that the amount of vanadium released from Ti alloy in TADs is well below the daily alimentary intake of this element [27,28].

### 4.2. Scanning Electron Microscopy (SEM) Investigation

SEM examination was performed to obtain a descriptive analysis and qualitative evaluation of the surface characteristics of the TADs, and identify the presence of any contaminants.

Anodic oxidation causes a slight increase in roughness; this is a micro roughness, compared to the macro roughness of alumina sandblasting [29,30]. The new miniscrews exhibited an irregular surface with machining and polishing defects in the form of stripes, which could represent electron points for electrochemical attacks. The used miniscrews exhibited indentation, corrosion attacks and cracks over the entire surface. The SEM images of the retrieved TADs did not exhibit any noticeable deformation, even though it has been demonstrated that corrosion and interactions with body fluids and tissues may significantly influence the surface morphological changes [29,30]. The loosening of TADs increases orthodontic treatment length and represents a negative factor for patient compliance. To control this inconvenience, digital solutions for TAD positioning could be considered a relevant alternative. In addition, the possibility of using stem cells offers new perspectives for controlling and modulating the inflammatory response in bone tissue and ensuring better stability [31,32].

Several research studies have been performed concerning the surface characteristics of TADs, but there are less examining the contamination of TAD surfaces when being manipulated. Additionally, clear information about the finishing of the screw surface is lacking [32,33,34,35,36].

When comparing SEM images, it can be surmised that the darkened areas on the inner threads of the mini-implants represent the oxidized surface (Figure 3, Figure 4 and Figure 5). SEM analysis indicates that the process of autoclave sterilization does not alter the mini-implant surface, as no defects in the form of pores or cracks and no images suggestive of corrosion could be identified in the autoclaved mini-implants.

Elides et al. found morphological and structural changes in the retrieved mini-implants, but no material structural changes in the form of defects or pores were documented [10]. In this study, too, no defects in the form of pores or cracks and no images suggestive of corrosion of this kind could be visualized in the retrieved mini-implants.

### 4.3. Microbiological Analysis

TADs are inserted in attached gingiva, and oral hygiene is considered a critical factor in mini-implant success. Acute and chronic inflammation induced by plaque retention can engender mobility and loss of the orthodontic mini-screw. Brushing teeth only is not sufficient in patients undergoing orthodontic treatment; antiseptic mouthwash containing chlorhexidine, essential oils, and povidone-iodine have been recommended in order to successfully reduce the microbiota, improving oral hygiene and consequently increasing the long-term success of mini-implants [37,38].

*S. Mutans* and *P. Gingivalis* were included, because they are plaque constituents, representing the initiation of periodontal inflammation [21,36].

After TAD insertion, a new site for microbial colonization is created that is comparable with a gingival sulcus between the attached gingiva and the transmucosal neck of the mini-implant [19,37,38]. This area remains in tight contact with the adjacent tissues, offering limited access and, consequently, making it difficult to clean [39,40,41]. Per-implant inflammation can be determined on the basis of microbial colonization, plaque retention device mobility, swelling, and loss of supporting bone mediated by the host’s immune and inflammatory responses [42].

Inflammation of the tissue surrounding TADs produces an increase in orthodontic mini-implant failure by 30%, but the failure mechanisms are still unclear [43,44].

The biocompatibility of orthodontic miniscrews is of principal concern, because they are inserted directly into the periodontal tissues and alveolar bones. Patients may have negative reactions, such as inflammation or necrosis of the oral mucosa, gingiva, or alveolar bone, to these miniscrews [40,42]. These reactions are highly correlated with the stability of the TiO_2_ film layer. Additionally, metallic ions released from orthodontic miniscrews can affect these reactions, and therefore, the contamination of orthodontic miniscrews from undesirable metals should be studied [19,40,41,42,43,44]. Bacterial contamination of TADs can have a significant impact on patient oral health. Bacterial adhesion onto the metal surface is influenced by the type of bacteria, the nature of the metal substrate, and the hydrophobicity of the material, as well as the energy of the surface [45,46,47].

Studies on biofilms fixed on metal substrates have been confirmed by results obtained for other types of materials, proving that the antibacterial activity of disinfectants used against these biofilms differs depending on the type of surface on which the bacteria attach and grow [45,48]. There are ethical considerations surrounding the re-use of invasive medical devices in different patients, despite the advantages with respect to costs. TADs may be used again in the same patient if the structural integrity and mechanical properties are not altered following their previous use, and following sterilization [49].

## 5. Conclusions

Within the limitations of this study, the following conclusions can drawn. The time, type of alloy and type of storage medium influenced the corrosion performance of orthodontic miniscrews—the null hypothesis was confirmed. TAD interactions under the specific conditions of the oral cavity must be considered by orthodontists in order to minimize failure rate. From a clinical perspective, these results can assist practitioners in the decision to re-use failed TADs in the same patient.

## Data Availability

The data presented in this study are available on request from the corresponding author.
